# Assessment of Left Ventricular Structural Remodelling in Patients with Diabetic Cardiomyopathy by Cardiovascular Magnetic Resonance

**DOI:** 10.1155/2016/4786925

**Published:** 2016-06-22

**Authors:** Yongning Shang, Xiaochun Zhang, Liu Chen, Weiling Leng, Xiaotian Lei, Qi Yang, Ziwen Liang, Jian Wang

**Affiliations:** ^1^Department of Radiology, Southwest Hospital, Third Military Medical University, Chongqing 400038, China; ^2^Department of Endocrinology, Southwest Hospital, Third Military Medical University, Chongqing 400038, China; ^3^Department of Radiology, Xuanwu Hospital, Beijing 100053, China

## Abstract

*Background*. Diabetic cardiomyopathy (DCM) is always accompanied with alteration of left ventricular structure and function. The aims of this study were to assess the structural remodelling in patients with DCM by cardiovascular magnetic resonance (CMR) and correlation of structural remodelling with severity of DCM.* Methods*. Twenty-five patients (53.8 ± 8.8 years, 52.0% males) with DCM and thirty-one normal healthy controls (51.9 ± 13.6 years, 45.2% males) were scanned by CMR cine to assess function and structure of left ventricular. Length of diabetic history and results of cardiac echocardiography (E′, A′, and E′/A′) were also measured.* Results*. Compared with normal controls group, DCM group was associated with significantly increased ratio of left ventricular mass at end diastole to end-diastolic volume (MVR) (*P* < 0.05) and no significant difference was in mass at end diastole (*P* > 0.05). The ratio correlated with both length of diabetic history and echocardiographic Doppler tissue imaging E′ (all *P* < 0.05).* Conclusions*. CMR can be a powerful technique to assess LV remodelling, and MVR may be considered as an imaging marker to evaluate the severity of LV remodelling in patients with DCM.

## 1. Introduction

Type 2 diabetes mellitus (T2DM), one of the most common chronic diseases, affects nearly four hundred million people in all countries [1]. As estimated [2], there will be a seventy-percent increase in adults with T2DM in developing countries and a nearly 20% increase in developed countries between 2010 and 2030. Diabetic cardiomyopathy is considered as myocardial dysfunction in almost two-thirds of patients with T2DM, which occurred independently of coronary heart disease (CHD), valvular dysfunction, and hypertension [3, 4]. The reasons for diabetes-related adverse myocardial alteration are not clear. But LV remodelling, especially concentric remodelling, is an emerging candidate mechanism. LV concentric remodelling, a kind of patterns of LV remodelling, is characterized by an increased ratio of LV mass to volume (MVR) and normal LV myocardial mass index at ED [5].

For a long time, LV remodelling has been assessed by echocardiography [6–8]. But there are several limitations for echocardiography, such as limited view and susceptible reproducibility, when compared with cardiovascular magnetic resonance (CMR). With high resolution and signal to noise ratio, CMR is being regarded as the golden standard for the assessment of cardiac function and structure [9, 10]. Importantly, the accuration and reproducibility of volume and mass measurement were well tested in inter- and intracenter [11].

There has not been widely accepted in normal range of MVR measured by CMR, let alone in patients with DCM. The special aims of this study were to investigate the LV remodelling in DCM patients by CMR and correlating between LV remodelling and diabetic history.

## 2. Patients and Methods

### 2.1. Patients

Twenty-five patients with diabetic cardiomyopathy were recruited from June 2015 to March 2016 at Southwest Hospital of Third Military Medical University. The inclusion criteria included (a) first diagnosis of type 2 diabetes mellitus with the criteria from World Health Organization [12], (b) decreased left ventricular diastolic function, the ratio of early diastolic mitral annular velocity E′ to late velocity A′ (E′/A′ < 1) by echocardiographic Doppler tissue imaging [13], and (c) no history of hypertension and suspicious coronary heart disease. The following were exclusion criteria: (a) estimated glomerular filtration rate (eGFR) ≤ 30 mL/min/1.7 m^2^, (b) age < 30 years, or >70 years, and (c) other standard CMR contraindications, such as claustrophobia or any history of body metal implant. Two candidates quitted because of high heart rate during being scanned. Therefore, a total of 25 patients were enrolled. At the same time, thirty-one age- and gender-matched normal healthy controls from community were recruited by their own volition to this study. All patients and normal controls were gave the informed consent, which were conducted in accordance with the Declaration of Helsinki (1964). This study was approved by the Institutional Review Board of Southwest Hospital (the first affiliated hospital of Third Military Medical University).

### 2.2. Demography Exam

All DCMs and normal controls were measured height, weight to yield body surface area (BSA) and body mass index (BMI), and systolic and diastolic blood pressure. Serum glucose, HbA1c, pancreas function (insulin, c peptide), renal function (blood urea nitrogen (BUN), creatinine, and cystatin C), lipids profile (triglycerides, total cholesterol, high-density and low-density lipoprotein cholesterol (HDL, LDL)) were analyzed. Myocardium zymogram examination (aspartate aminotransferase (AST), lactate dehydrogenase (LDH), *α*-hydroxybutyrate dehydrogenase (*α*-HBDH), creatine kinase (CK), ischemia modified albumin (IMA)), and markers of myocardial damage (troponin and myoglobin) were assessed in 86% patients and controls. Urine microalbumin (U-MTB) was also measured in the majority of participants (79%).

### 2.3. Echocardiography

All DCMs were performed transthoracic echocardiography at rest by a commercial ultrasound machine (Philips IE33 color Doppler scanner, the Netherlands) and a matching transducer (S5-1 heart probe). According to the American Society of Echocardiography's recommended guidelines [13], assessment of LV diastolic function was carried out by the cardiac echocardiographic Doppler tissue imaging (DTI) at an apical four-chamber view with the sample volume placed at mitral valve annulus. Early diastolic septal mitral annular velocity E′ (passive left ventricular filling), late velocity A′ (atrial contraction), and their ratio (E′/A′) were measured to yield the LV diastolic function. All images were stored in Digital Imaging and Communications in Medicine (DICOM) format for online analysis.

### 2.4. Cardiovascular Magnetic Resonance Protocol

All DCMs and healthy controls were scanned by CMR on a 3.0 T whole-body system (Trio Tim, Siemens Healthcare, Erlangen, Germany) in the supine position and head first with a matching 12-channel matrix body coil and electrocardiographic gating instrument. Breath held in expiration when necessary. The localizing images, including standard four-chamber (left ventricular, left atrium, right ventricular and right atrium), two-chamber (left ventricular and left atrium), and short-axis images, were obtained.

Segmented short-axis cine images were acquired with two-dimensional (2D) steady-state free precession (SSFP) pulse sequence (TR/TE 59.22/1.45 ms, FOV 400 × 325 mm^2^, flip angle 50°, matrix size 256 × 179, voxel size 2.2 × 1.6 × 6.0 mm^3^, slice thickness 6 mm, spacing between slices 1.5 mm, and 25 phases per cardiac cycle) [14]. To cover the whole LV, the position line of the first basal cine images was localized through both the insertion point of mitral valve into septum and free wall in both standard four-chamber and two-chamber slice. The last short-axis cine was localized at apex. The position lines of all other slices were put to parallel to the first line. Because of difference in length of left ventricular among all participants, the number of their own slices ranged from 8 to 14. Similarly, due to difference in heart rate among them, time of holding breath ranged from 6 to 10 seconds.

### 2.5. Cardiovascular Magnetic Resonance Analysis

**Figure 1:**
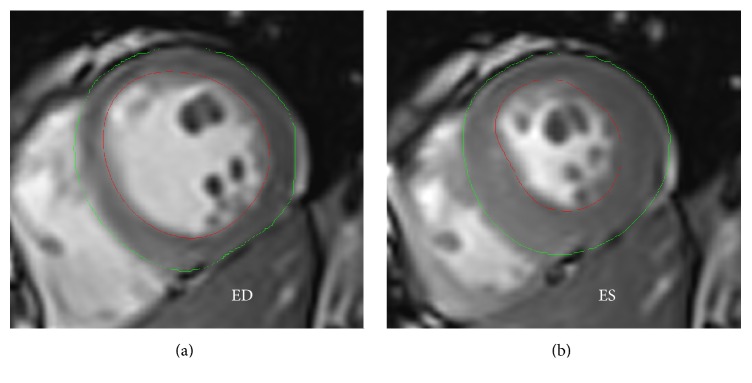
Epi- and endocardial contours on end-diastolic phase (a) and end-systolic phase (b).

Cine images were analyzed offline using the dedicated commercial available software (Argus, Siemens Healthcare, Erlangen, Germany) by two experienced observers (one with 10 years of experience in CMR and the other 6 years of experience) without knowing any information of the participants. After all short-axis cine images of LV were loaded, the end-systolic (ES) phase and end-diastolic (ED) phase were visually identified as the smallest and largest chamber area separately on the middle slice. The myocardial endocardial and epicardial contours of LV were carefully manually delineated on all images (Figure 1) to acquire the absolute function parameters including end-systolic volume (ESV), end-diastolic volume (EDV), stroke volume (SV), ejection fraction (EF), cardiac output (CO), myocardial mass at ED, and normalized function parameters including ESV/BSA (ESVi), EDV/BSA (EDVi), SV/BSA (SVi), cardiac index (CI), myocardial mass/BSA (massi), and ratio of myocardial mass to volume of LV [15]. Moderator bands and papillary muscles were carefully assigned in the lumen of LV.

### 2.6. Statistical Analysis

Categorical variables were showed as percentage. Kolmogorov-Smirnov test was used to test normality of variables, and variables that did not fit normal distribution were summarized as median and quartile. Variables, which were continuously and normal distribution, were presented as mean and standard deviation (SD). Independent sample *t*-test was used to evaluate the differences in function parameters and demographic variables between the DCMs and normal. Mann-Whitney *U* test was used to evaluate the differences in biochemical variables if the variables did not fit normal distribution or standard variance was heterogeneity. The relationship between MVR and diabetic history was analyzed by Pearson's or Spearman's method. Statistical tests were two-tailed, and *P* < 0.05 was considered to reach statistical significance. Statistical analysis was performed on the commercial available software (SPSS for Windows 21.0, Inc, Chicago, IL, USA).

## 3. Results

### 3.1. Results of Demographic and Biochemical Characteristics

Results of demographic and biochemical characteristics were showed in Table 1.

Twenty-five patients (13 male, age 53.8 ± 8.8 years, diabetic history 5.9 ± 3.9 years) with DCM, and thirty-one normal controls (14 male, age 51.9 ± 13.6 years) were studied. Fasting blood glucose and glycated hemoglobin significantly increased in DCMs. Insulin and C peptide were also elevated in DCMs. DCMs were also accompanied by higher triglycerides, HDL, and aspartate aminotransferase (AST). IMA and troponin were higher in DCMs, which showed possible impairment of myocardium related to T2DM. Age, gender, weight, height, BSA, BMI, blood pressure, and renal function (BUN, creatinine, cystatin C) were similar in the two groups (all *P* > 0.05).

### 3.2. Alteration of Left Ventricular Geometry and Function

Parameters of LV function and structure were summarized in Table 2.

**Figure 2:**
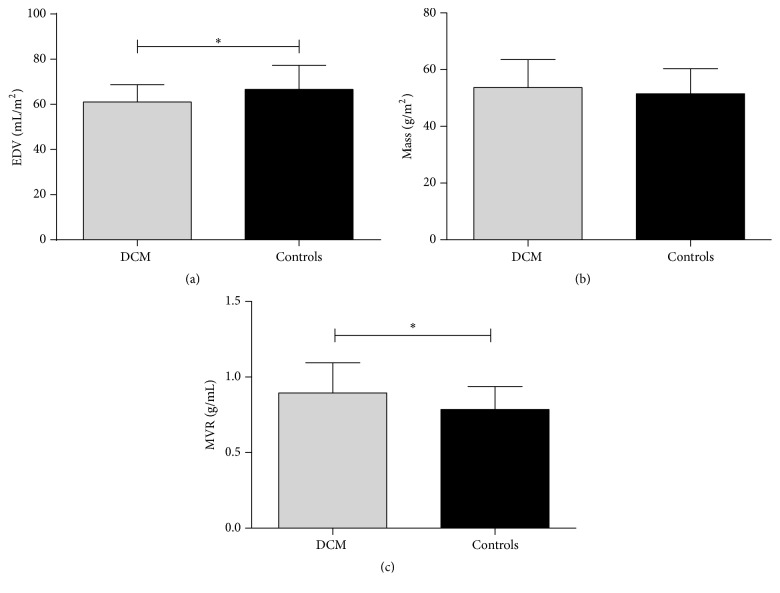
Differences in left ventricular geometry between DCMs and controls: (a) EDV, (b) myocardial mass at ED, and (c) MVR. *∗* means that *P* < 0.05.

The LV EDVi approximately decreased by 10% in DCMs (60.1 ± 7.7 versus 66.6 ± 10.6 mL/m^2^, *P* = 0.032). There was no significant difference in LV massi at ED between the two groups (53.7 ± 9.8 versus 51.5 ± 8.9 g/m^2^, *P* = 0.378). MVR increased by 15% in DCMs (0.90 ± 0.20 versus 0.79 ± 0.15 g/mL, *P* = 0.025; Table 2, Figure 2), suggesting that there was significant LV concentric remodelling. MVR did not show correlation with systolic blood pressure, diastolic blood pressure, age, height, weight, BMI, or BSA by separately bivariate Pearson's correlation test (All *P* > 0.05).

The LV ESVi was also smaller in DCMs (24.6 ± 5.9 versus 28.6 ± 6.9 mL/m^2^, *P* = 0.023). DCM patients were similar in SVi, CI, and EF to normal controls (All *P* > 0.05), which revealed that systolic function of LV did not impair in DCMs. Echocardiography DTI showed that DCMs were associated with decreased E′/A′ (mean 0.80 ± 0.09).

### 3.3. The Relationship between Concentric Remodelling and Diabetic History

**Figure 3:**
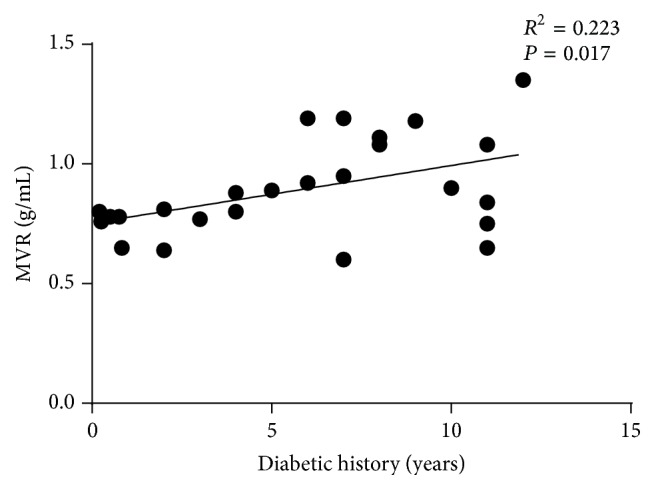
Relationship between length of diabetic history and MVR.

**Figure 4:**
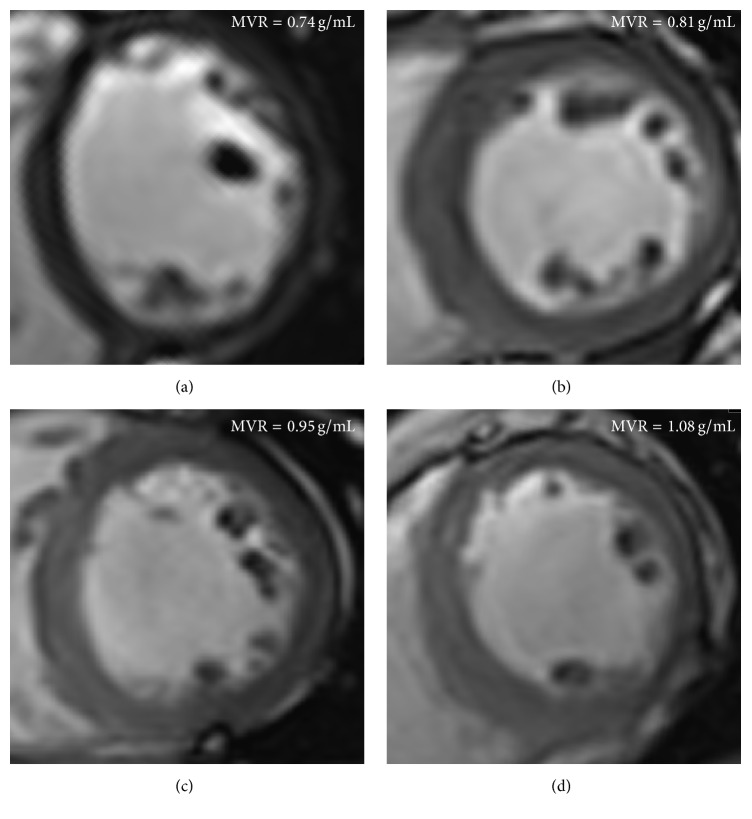
Representative examples of cardiac cine images in control and DCMs. (a) Control, 63 years old, female, EDVi = 69.9 mL/m^2^, massi = 51.4 g/m^2^, MVR = 0.74 g/mL. (b) DCM (2 years), 60 years old, male, EDVi = 63.1 mL/m^2^, massi = 51.0 g/m^2^, MVR = 0.81 g/mL. (c) DCM (7 years), 40 years old, male, EDVi = 66.3 mL/m^2^, massi = 63.0 g/m^2^, and MVR = 0.95 g/mL. (d) DCM (11 years), 57 years old, EDVi = 53.7 mL/m^2^, massi = 58.0 g/m^2^, and MVR = 1.08 g/mL.

Pearson correlation analysis showed that MVR was positively correlated with the length of diabetic history (*r* = 0.472, *P* = 0.017, Figures 3 and 4). Stepwise multivariable linear regression showed that the length of diabetic history was the only independent predictor of LV MVR (normalized *β* = 0.472, *P* = 0.017; demographic variable, age, height, weight, BMI, BSA, and systolic and diastolic blood pressure). Spearman correlation showed that MVR was negatively correlated with echocardiography DTI E′ (*ρ* = −0.435, *P* = 0.030), although the correlation between MVR and E′/A′ did not reach statistical significance.

## 4. Discussion

Diabetic cardiomyopathy is regarded as T2DM-related myocardial dysfunction, which is independently of CHD, valvular dysfunction, and hypertension [3, 4]. In agreement with previous reports [16, 17], DCM was accompanied by changed LV geometry and function. Using CMR cine we show here that (1) DCM is accompanied by LV concentric remodelling, (2) MVR is associated with length of diabetic history, and (3) MVR is correlated with cardiac echocardiography DTI E′, indicating impaired LV diastolic function.

As reported in previous study [18–20], there is insulin resistance in patients with T2DM, in other words, pancreas islet secretes more insulin and C peptide, which causes hyperinsulinemia. In our study, DCMs were associated with hyperinsulinemia, higher C peptide, and metabolic syndrome, manifesting increased triglycerides and decreased HDL. IMA, as a novel biomarker of tissue ischemia, was higher in diabetic patients than normal controls [21, 22], and elevated IMA levels also predict a subclinical cardiovascular disease in T2DM [23–25]. Our study showed that patients with DCM were associated with increased IMA, which indicated that myocardial tissue might suffer T2TM-related tissue ischemia. Our research was consistent with previous studies. Troponin, a biomarker of myocardial damage, are widely used to evaluate situation of patients with acute coronary syndromes. Nowadays, large cohort study [26] and case-control study [27] considered the cardiac troponin as independent predictor of major adverse cardiovascular events in T2DM patients. In this study, increased troponin parameters were found in patients with DCM.

LV concentric remodelling, a kind of patterns of LV remodelling, was characterized by an increased ratio of LV mass to volume and normal LV myocardial mass index at ED [5]. For a long time, LV remodelling has been assessed by echocardiography. Due to limited view and susceptible reproducibility, echocardiography might not be a powerful technique when compared with CMR. CMR, with high spatial resolution and signal to noise ratio, is widely used to assess cardiac function and structure and being regarded as the golden standard. Therefore, CMR may be the better technique to assess MVR than echocardiography. Many kinds of disease may cause LV concentric remodelling. Previous studies have reported diabetes-related LV concentric remodelling [16, 28]. In our study age- and gender-matched normal healthy controls, in the absence of CHD, hypertension, and other cardiovascular disease, were enrolled in the same time. In this study, MVR of normal group was 0.79 ± 0.15 g/mL, which was consistent with previous studies with large population (totally 741 subjects) [29] and small population [16]. However, it is smaller than the value of MVR derived from another small study [28] and multiethnic study of atherosclerosis [30]. This is like the fact that the population, enrolled in the latter large study, was not highly selected. Participants with hypertension and even diabetes were included; therefore the results should not be considered as real range of healthy subjects. So large study with highly selected normal healthy population should be conducted to confirm the values of MVR in healthy subjects. The values of MVR in patients with DCM were not well-defined. In this study, MVR of DCM group was 0.90 ± 0.20 g/mL, which is consistent with the value of the study [16], but a little smaller than the value of the study [28]. Both the two studies enrolled relatively small objects. Considering the heterogeneity of T2DM, large and highly selected patients should be recruited to discover the mystery of MVR in DCM.

It is intriguing that MVR was correlated with length of diabetic history, not age, blood pressure, changed AST, IMA, triglycerides, HDL, insulin, C peptide, glucose, and even HbA1c. Transient hyperglycemia can make persistent impairment to cell by disrupting signal feedback loop [31]. HBA1c, biomarker of time-averaged glucose level, and hyperglycemia cannot manifest the severity of long-term accumulated damage to myocardium. Neither the elevated troponin nor IMA can manifest the severity of long-term accumulated damage to myocardium. The relationship between length of diabetic history and MVR may prompt us that MVR may be considered as a biomarker of long-term effect of T2DM to myocardium, which should be confirmed by large cohort or cross section studies. What is more, decreased cardiac echocardiography DTI E′ and E′/A′ have been widely accepted as the marker of impaired LV early diastolic function [13]. In this study, we also found that E′ was correlated with MVR. Both of them indicated us that MVR of LV increased with diabetic progression, shown as length of diabetic history and impaired LV diastolic function. Taking together, MVR may be considered as an imaging marker to assess the severity of DCM.

There were some limitations in our study. First, the number of recruited patients and normal controls was small, and this study should be considered as preliminary. Large cohort or cross-section studies should be conducted to confirm the influences of diabetic history on LV remodelling. Second, patients only with GFR ≥ 30 mL/min/1.7 m^2^ were recruited, and this inhomogeneity of patients may introduce a bias.

## 5. Conclusion

CMR can be a powerful technique to assess LV remodelling, and MVR may be considered as an imaging marker to evaluate the severity of LV remodelling in patients with DCM.

## Figures and Tables

**Table 1:** Demographic characteristics.

	DCM (*N* = 25)	Control (*N* = 31)	*P* value
Anthropometry			
Age, y	53.8 ± 8.8	51.9 ± 13.6	0.533
Diabetic history, y	5.9 ± 3.9		
Male, %	52.0	45.2	0.611
Height, m	1.62 ± 0.07	1.61 ± 0.07	0.634
Weight, kg	63.8 ± 11.6	61.0 ± 10.5	0.336
BMI, kg/m^2^	24.1 ± 2.9	23.4 ± 3.1	0.362
BSA, m^2^	1.68 ± 0.18	1.65 ± 0.17	0.457
Systolic blood pressure, mmHg	122.2 ± 10.0	119.3 ± 11.5	0.324
Diastolic blood pressure, mmHg	81.6 ± 6.4	83.5 ± 7.7	0.333

Biochemical exam			
Urine microalbumin, mg/dL	0.3 [0.1–5.0]	1.2 [0.6–1.8]	0.177
Blood urea nitrogen, mmol/L	5.8 [4.8–7.0]	6.0 [5.1–6.9]	0.716
Creatinine, umol/L	68.7 ± 21.9	64.1 ± 16.9	0.381
Cystatin C, mg/L	0.77 [0.66–0.88]	0.75 [0.64–0.86]	0.594
AST,	22.0 [17.8–29.5]	29.5 [23.2–35.4]	**0.008**
LDH, IU/L	201.0 ± 45.3	197.7 ± 46.5	0.809
*α*-HBDH, IU/L	128.4 ± 31.6	131.2 ± 38.7	0.799
CK, IU/L	97.4 [69.9–123.5]	86.0 [65.4–108.4]	0.421
Ischemia modified albumin, U/mL	81.9 ± 7.5	73.6 ± 5.9	**0.001**
Total cholesterol, mmol/L	5.7 [4.8–6.9]	5.3 [4.9–6.0]	0.205
Triglycerides, mmol/L	2.4 [1.5–4.3]	1.3 [0.9–2.0]	**0.004**
HDL, mmol/L	1.2 [1.0–1.4]	1.4 [1.1–1.7]	**0.026**
LDL, mmol/L	3.5 [2.8–4.3]	3.3 [3.1–3.7]	0.703
Glucose, mmol/L	8.6 [7.2–11.1]	5.3 [4.9–5.8]	**0.000**
Glycated hemoglobin, %	6.9 [6.3–9.1]	5.6 [5.4–5.8]	**0.000**
Troponin, 10^−3^ ug/L	8 [5–12]	5 [3–6]	**0.003**
Myoglobin, ng/mL	27.6 [22.3–49.4]	30.5 [22.4–38.5]	0.739
Insulin, uIU/mL	16.4 ± 7.0	12.1 ± 4.3	**0.022**
C peptide, ng/mL	1.7 [1.2–2.3]	1.1 [0.8–1.4]	**0.007**

Echocardiography			
Doppler mitral annular velocity E′/A′	0.80 ± 0.09		

**Table 2:** Left ventricular geometry and function.

	DCM (*N* = 25)	Controls (*N* = 31)	*P* value
LV end-diastolic volume index (EDV), mL/m^2^	60.1 ± 7.7	66.6 ± 10.6	**0.032**
LV end-systolic volume index (ESV), mL/m^2^	24.6 ± 5.9	28.6 ± 6.9	**0.023**
LV stroke volume index (SV), mL/m^2^	36.5 ± 4.7	38.0 ± 5.4	0.279
LV ejection fraction, %	60.1 ± 6.7	57.5 ± 5.3	0.102
Cardiac index (CI), L/(min*∗*m^2^)	2.8 ± 0.4	2.7 ± 0.5	0.749
LV mass index, g/m^2^	53.7 ± 9.8	51.5 ± 8.9	0.378
LV mass to LV end-diastolic volume, g/mL	0.90 ± 0.20	0.79 ± 0.15	**0.025**
